# Supplementary material for: The Spread of Lone Star Ticks (Amblyomma americanum) and Persistence of Blacklegged Ticks (Ixodes scapularis) on a Coastal Island in Massachusetts, USA
Source: Insects. 2024 Sep 17;15(9):709. doi: 10.3390/insects15090709 (PMC11432020; doi:10.3390/insects15090709)
Supplement: Supplementary file 1 [file insects-15-00709-s001.zip › insects-3183494-supplementary.pdf]

Supplementary Table S1. Numbers of yard surveys carried out in each town on May 15 – July 31, from 2011-2024. Chappaquiddick Island, which is part of Edgartown, is listed separately.

	2011	2012	2013	2014	2015	2016	2017	2018	2019	2020	2021	2022	2023	2024	All years
Aquinnah	0	0	0	0	0	6	10	10	9	3	3	5	8	2	56
Chappaquiddick	29	67	0	82	31	10	10	28	45	3	24	8	10	9	356
Chilmark	0	7	53	58	22	36	17	44	33	15	9	9	25	17	345
Edgartown	0	0	0	0	1	20	11	29	22	5	8	28	25	19	168
Oak Bluffs	0	0	0	0	0	13	6	14	12	2	1	5	12	23	88
Vineyard Haven	0	0	0	0	0	19	6	23	11	3	4	14	19	3	102
West Tisbury	0	0	0	0	0	8	13	32	23	7	10	20	18	19	150
All towns	29	74	53	140	54	112	73	180	155	38	59	89	117	92	1265

Supplementary Table S2. Percent of each trail at wooded sites with branches overhead, and percent with branches of each tree species overhead. N is the number of evenly spaced observation points along the trail (see Methods). Many observation points had branches of more than one tree species overhead. At one site, Jeremiah Woods, 20 percent of the observation points did not have shade (no branches overhead and no high shrubs along the trail edge), while other sites were more shaded.

Site ID	Sample size	Branches overhead	No branches or high shrubs	*Pinus rigida*	*Quercus velutina*	*Quercus alba*	*Sassafras albidum*	*Amelanchier spp.*	*Carya spp.*	*Quercus ilicifolia*	*Nyssa sylvatica*	*Prunus serotina*	*Fagus grandifolia*
Packard	N=47	96	4	66	40	6	17	4		6		2	
Jeremiah	N=30	80	20	53	17	20	3	3		3			
Mytoi	N=44	98	2	48	70	50	7	2			9		
Three Ponds	N=30	100	0	40	60	23	0						
Sampson	N=34	100	0	9	62	41	24		6				
Pocha	N=38	84	3	3	37	55	8	3				3	
Slater	N=50	90	4	0	66	46	26	4					6
Ames	N=30	100	0	0	60	70	60	33	3				
Tilghman	N=38	87	0	0	3	24	8	5	63	3	3		

Supplementary Table S3. Average tick densities per 0.5 km at wooded sites in 2023 and 2024. N = 5 sampling days per site (except Slater, where N = 4). BL = blacklegged tick (*Ixodes scapularis*), LS = lone star ticks (*Amblyomma americanum*). Lone star "larval clusters" refers to number of 12 m sweeps per 0.5 km with $\geq$ 50 larvae.

Site	Year	BL Nymphs	BL Adults	LS Nymphs	LS Adults	LS Larval Clusters
**ALL SITES**	**2023**	**63**	**1**	**33**	**11**	**0**
Mytoi	2023	63	0	44	9	1
Packard	2023	72	2	32	22	1
Tilghman	2023	67	3	29	11	0
Slater	2023	69	0	43	11	0
3 Ponds	2023	44	1	15	3	0
**ALL SITES**	**2024**	**68**	**2**	**51**	**32**	**1**
Mytoi	2024	33	0	20	9	1
Packard	2024	52	3	47	24	5
Tilghman	2024	107	3	78	15	0
Jeremiiah	2024	52	5	36	32	0
Ames	2024	118	2	87	43	0
Sampson	2024	64	2	52	40	1
Pocha	2024	51	2	37	59	1
